# Using word embeddings to investigate cultural biases

**DOI:** 10.1111/bjso.12560

**Published:** 2022-07-23

**Authors:** Kevin Durrheim, Maria Schuld, Martin Mafunda, Sindisiwe Mazibuko

**Affiliations:** ^1^ Psychology University of Johannesburg Johannesburg South Africa; ^2^ School of Chemistry of Physics University of KwaZulu‐Natal Durban South Africa; ^3^ Discipline of Psychology University of KwaZulu‐Natal Pietermaritzburg South Africa

**Keywords:** attitudes, bias, implicit meaning, stereotypes, word embeddings

## Abstract

Word embeddings provide quantitative representations of word semantics and the associations between word meanings in text data, including in large repositories in media and social media archives. This article introduces social psychologists to word embedding research via a consideration of bias analysis, a topic of central concern in the discipline. We explain how word embeddings are constructed and how they can be used to measure bias along bipolar dimensions that are comparable to semantic differential scales. We review recent studies that show how familiar social biases can be detected in embeddings and how these change over time and in conjunction with real‐world discriminatory practices. The evidence suggests that embeddings yield valid and reliable estimates of bias and that they can identify subtle biases that may not be communicated explicitly. We argue that word embedding research can extend scholarship on prejudice and stereotyping, providing measures of the bias environment of human thought and action.

## BACKGROUND

A bias is a ‘subjectively based preference’ for one conclusion or inference over alternatives (Kruglanski & Ajzen, 1983, p. 19). Group prejudices may be expressed as biased evaluations and stereotyped beliefs such as ‘women are emotional’, ‘scientists are male’ or ‘fat people are lazy’. Individuals may hold these biases implicitly as prejudgments or express them as explicit attitudes (Devine, 1989). A great deal of research has studied biases in minds of individuals (Paluck et al., 2021), but is it possible to study social biases in the large volumes of text produced in media, social media and other interactions? This article will show how word embeddings can be used to uncover prejudices and stereotypes that may be expressed explicitly or implicitly in social and cultural discourse.

Implicit and explicit biases have traditionally been measured as responses to representations communicated in language. Attitude scales and opinion surveys present individuals with a target word or opinion statement and ask respondents for their evaluations along semantic dimensions such as ‘good’ versus ‘bad’ or by indicating their levels of agreement or disagreement (Osgood et al., 1957). Rather than eliciting explicit agreement or disagreement, measures of implicit bias record reaction times to estimate whether group primes (e.g., pictures of faces of black or white people) facilitate or inhibit recognition of positive or negative words (e.g., good, bad, love and hate; Greenwald et al., 2002). In all this work, language is treated as an instrument to measure mental biases, not as the source or medium of bias itself.

A large experimental and qualitative literature has investigated how biases are constructed and communicated in the language (Beukeboom & Burgers, 2019; Durrheim, 2021). First, words carry biased connotations which may be communicated even in ostensibly descriptive talk (cf. Maass, 1999; Potter, 1996). Second, individuals use these affordances of language to express opinions, signal their group identity and perform other collaborative actions (Kervyn et al., 2012; McGarty et al., 2009; Wetherell, 1998). This work shows that people express biases in creative, strategic and flexible ways that are not readily captured in the language of attitude measures (Potter & Wetherell, 1987). For example, the policy of the UK Home office that ‘unaccompanied asylum seeking children’ should ‘receive the necessary services and support’ conveys both the idea of sympathetic care of children and the distrust of asylum seekers, implicitly raising concerns about ‘illegal immigrants’ seeking to exploit the system (Aldridge‐Deacon & Aldridge, 2022).

Experimental and qualitative studies of language bias share the limitation of being labour intensive, with each study thus restricted to a very small set of words, concepts, or instances of talk. Advances in computing technology have facilitated the development of ‘systematic and reliable technique[s]’ for automating content analysis (Stemler, 2000). These make it possible to study biases as they are produced in everyday interaction in large volume in media, social media, scientific and other discourses. Computerized text analysis methods like the Linguistic Inquiry and Word Count (LIWC) use validated dictionaries of word meanings to count word frequencies (e.g., pronouns, social group markers and verbal aggression), and identify psychologically meaningfully categories of text reflecting emotions, thinking styles and so on (Tausczik & Pennebaker, 2010). New automated methods combine machine learning and automated content analysis to identify online intergroup prejudice (Dutta et al., 2018) and to mine opinions, sentiments and stances (ALDayel & Magdy, 2021; Liu, 2012).

The objectivity of the computerized methods of content analysis comes at a cost, however. They rely on predefined word meanings housed in curated dictionaries which are insensitive to the shifting meanings that language users give to words in the context of the debate. Dictionary definition of words ‘cannot cope with vagueness, with polysemy, with metaphoric or connotative connections’. (Bruner, 1990, p. 5). A language user can convey implicit negative stereotypes about illegal immigrants while showing concern for ‘unaccompanied asylum seeking children’ but such localized subtle meanings would hardly feature in any dictionary. In the ordinary talk, the word *asylum*, itself, might take on very different meanings in talk about refugees from Africa or the war in Ukraine.

This article reviews the application of computerized word embedding methods to studies of social bias in text data. Like sentiment analysis, word embedding methods are able to process vast quantities of data in a systematic and reliable manner. However, word embeddings do not require pre‐defined dictionaries but instead are able to deduce the meaning and bias of words from their context of use. They are thus able to capture some of the subtle and local meanings that speakers use to communicate biases. In this article, we first explain what word embeddings are and describe how they encode biases. We then review key studies that have used embeddings to study social biases, showing the utility of these methods for social scientists. Finally, we reflect on the promises, prospects and challenges of using word embedding methods for studying social biases.

## WHAT ARE WORD EMBEDDINGS?

Word embedding analysis is a method of natural language processing that uses machine learning to train a neural network to predict the contexts within which words are used (Smith, 2020). The algorithm reads a large text universe such as Wikipedia or Google News, learning to predict the word which follows a small window of read words. What, for example, is the missing word in the sentence, ‘ICE arrests 128 illegal _______ in Calif’. (Shaw, 2020). The algorithm will have candidate words – for example, *immigrants*, *refugees* and *foreigners* – and will re‐adjust the weights of the candidates after learning the correct word, *immigrants*. As the algorithm proceeds, word by word, through millions of documents, sentences and word tokens, it learns the meanings of words from the company they keep in their contexts of use.^1^

A word embedding is a set of vectors produced by the training described above, which provides a quantitative model of word meaning based on how the words are used. Each vector in the set corresponds to one word and the entries (weights) of a vector – usually between *n* = 200 and *n* = 500 – give the word a unique numerical representation. Words are embedded in the sense that words that can be used interchangeably (e.g., *immigrants* and *refugees*) will have similar vectors of weights indicating their similarity of meanings. The embedding is a closed system in which the meaning of each word is defined with reference to its similarity to each other word in the embedding. Words that can be used interchangeably but are never used together (e.g., synonyms) will thus have similar assigned meanings because of their similar relationship with all other words in the embedding (see Kozlowski et al., 2019, p. 18).

**FIGURE 1:**
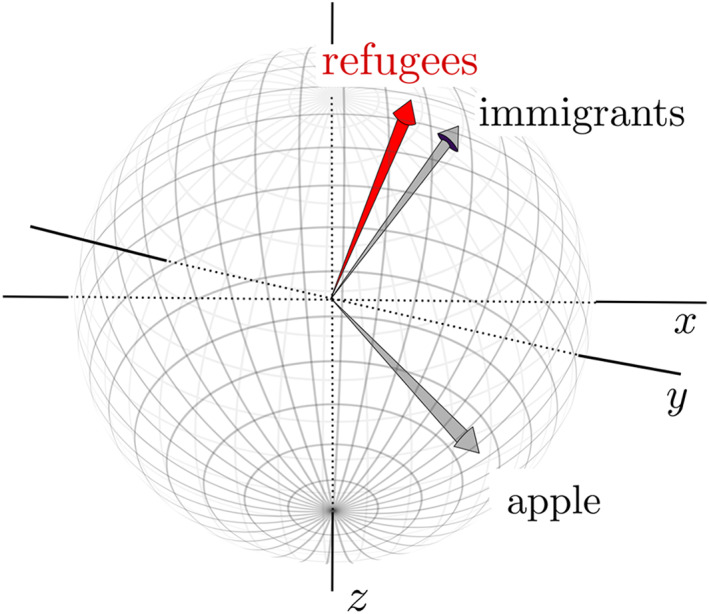
Spatial representation of a word embedding reduced to three‐dimension in which all vectors have been normalized to unit length. Each word in the embedding will be located at a unique address on the sphere and differences in word meaning can be gauged from the distance between word locations. Here, *immigrants* and *refugees* are embedded close to each other but positioned far from *apple*

Conveniently, the vector representation of a word embedding will have a spatial analogue. The word vectors define unique addresses for each word in the n‐dimensional hyperspace of the embedding. For illustrative purposes, we might use principal component analysis to reduce a 500‐dimensional word embedding to three dimensions, *x*, *y* and *z* (see Figure 1). We thus reduce the complexity of the n‐dimensional hyperspace to a three‐dimensional sphere that locates each embedded word at a unique address defined by its (normalized) weights in the three dimensions (see Figure 1). Words with similar meanings are found at similar addresses on the sphere and comparing these addresses would provide a quantitative index of how ‘related’ the words are. We could determine, for example, whether the word *immigrant* is closer to the word *illegal* or to the word *opportunity*, whether its meaning is closer to *black* or *white*, or whether it is closely related to the word *terrorist*. Of course, the reduction in the 500‐dimensional hyperspace to a three‐dimensional sphere would result in a loss of precision in meaning estimation.

## WHAT IS BIAS IN A WORD EMBEDDING?

Early research with word embeddings reported disturbing observations of social biases being encoded into the word meanings. Bolukbasi et al. (2016) found that the closest word to *black_male* in their embedding trained on Google News was *assaulted* while the closest bigram to *white_male* was *entitled_to*. Embeddings also encode doctors and computer programmers as male professions and nurses and homemakers as female professions (Ethayarajh et al., 2019). Caliskan et al. (2017, p. 1) argued that embeddings ‘absorb’ such biases in the process of training, where the algorithm learns to recognize stereotypical and biased associations of word meanings, which are ‘regularities latent in our culture’ and embedded in our language (ibid, p. 2). Word embeddings pick up these latent associated word meanings because they do not simply represent words that co‐occur, but they define the relations of each word to every other in the training data. Although crude attitudes and stereotypes are hardly ever articulated explicitly, the implicit associations can be identified from the positioning of the words in trained embeddings.

**FIGURE 2:**
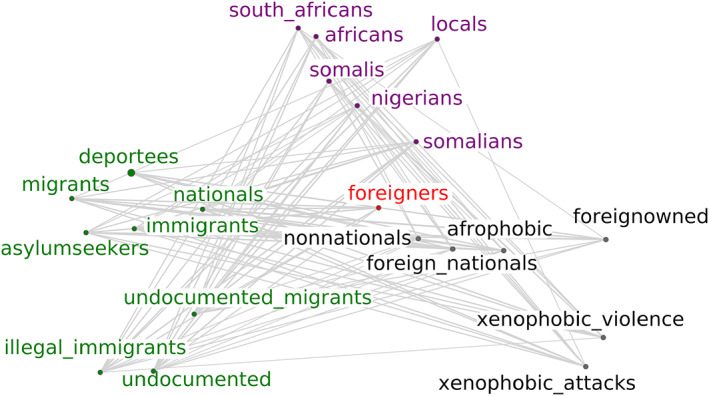
The 20 nearest neighbours of the word *foreigners* in a word embedding trained on South African newspaper articles

The implicit encoding of biases into word vectors is illustrated in Figure 2, which provides a network representation of the 20 nearest neighbours of the word *foreigners* in a word embedding trained on South African newspaper articles that use the word *foreigner*.^2^ The network reveals the structure of meanings surrounding the word *foreigners* from the context within which the word is used. The word is linked to violence and xenophobia, citizenship and concepts around legality, migration and asylum. It is also interesting that words like exotic, travel, meet and food are not among the nearest neighbours. By considering the structure and content of the nearest neighbours (and significant absences), we may begin to understand the biased way the word *foreigners* is used in the text corpus of South African news.

Data scientists are developing methods to de‐bias embeddings to produce culturally fair applications such as gender‐neutral job search engines (Bolukbasi et al., 2016; Ethayarajh et al., 2019; Zhao et al., 2019). Social scientists, by contrast, have used language biases in word embeddings to identify patterns of social stereotyping and prejudice. In the following two sections, we consider some of the methods they have used to measure (1) the strength of associations between word meanings, and (2) the degree to which concepts are evaluated negatively versus positively and associated with race, gender and other stereotypes. This work can help identify explicit and implicit biases in text archives and social media communications, understand how bias has evolved over time and in different contexts and show how these biases inform cultural practices of discrimination and inequality.

## MEANING ASSOCIATIONS IN WORD EMBEDDINGS

**FIGURE 3:**
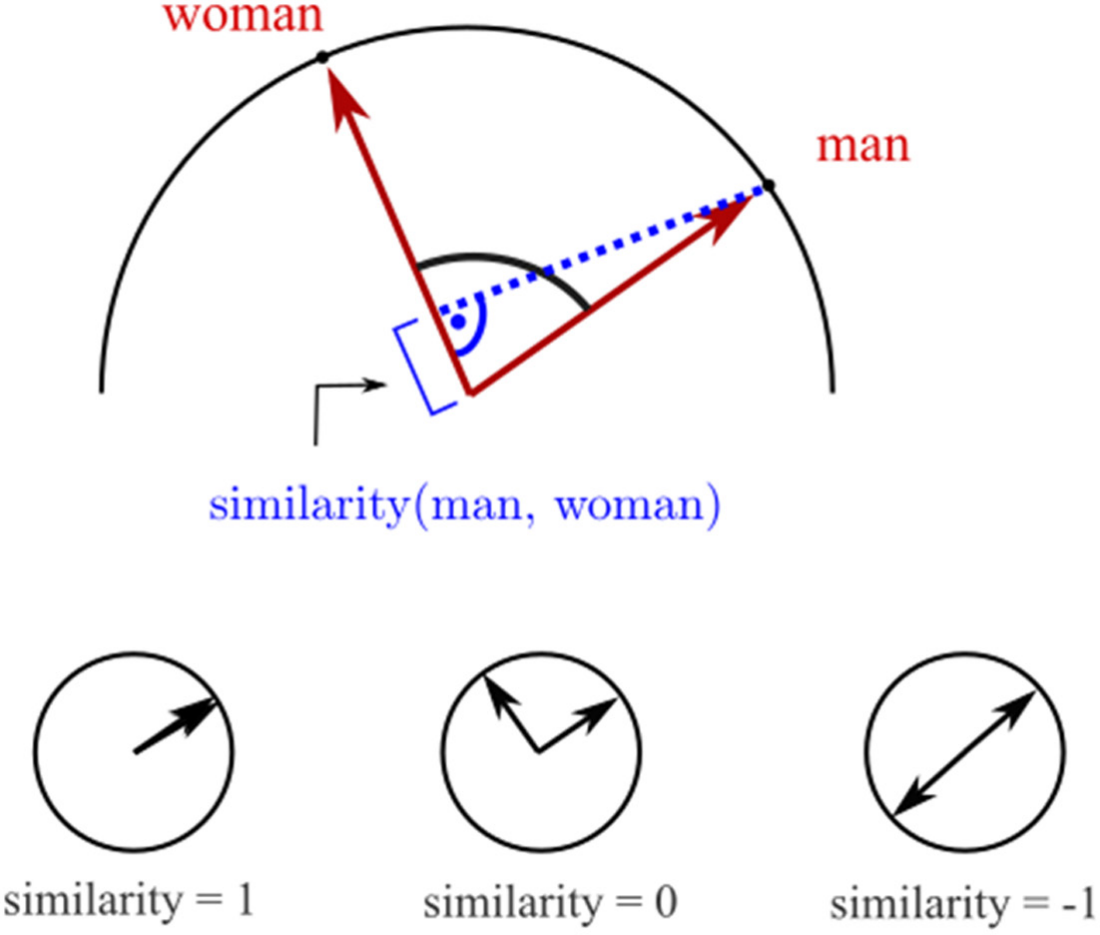
Cosine similarity as a measure of word meaning associations

Once a universe of words is arranged such that closely related words are positioned near each other in the embedding, the strength of association between word meanings is represented by the distance between the words. The cosine similarity score computes closeness by measuring the angle between word vectors. Refer back to Figure 1 to see how the angle between two vectors – for *refugee* and *immigrants* – can measure the distance between them. Cosine similarity scores range from −1 (= opposite meaning), through 0 (= no relation) to 1 (= identical meaning), and provide the simplest measure of shared meaning in an embedding (See Figure 3). Proximity measures of associations between concepts such as *woman* and *homemaker* (Bolukbasi et al., 2016) are the starting point for unearthing cultural associations that underlie social biases.

Rodman (2020) used the proximity method to analyse the changing meaning of the concept of ‘social equality’. She trained a *Word2Vec* model on 3105 New York Times articles that included the word ‘equality’ in the headline. She found that the proximity of *social* to *equality* decreased from 1855 to 1955, mirroring the declining euphemistic use of *social equality* to describe racial segregation. However, the proximity of *social* to *equality* increased again from 1955 to 2016 as the concept began to describe economic relationships in phrases such as *economic equality*, *social issues* and *social justice*. The associated meanings behind the idea of ‘social equality’ in US media discourse ‘inversely tracks racial progress’ (p. 109).

Whereas Rodman computed the closeness of two words over time, Stoltz and Taylor (2019) developed a method to compare documents by computing the closeness of all the words in each document to a target word. In one study, they investigated how closely all the words in different Shakespeare plays are aligned to the word *death* (Richard III was the deathliest). In another study, they analysed all 239 US State of the Union Addresses from 1790 to 2018 to investigate changing representations of authority. They found that early speeches were closer to the concept of *strict_father* than *nurturing_parent* but this changed over the twentieth century, especially since the 1950s, reflecting deep changes in culture.

This work is still in its infancy but has many potential applications for identifying biases in the language used by different speakers, groups or organizations, across topics and over time. Although large volumes of data are required to train reliable embedding models, the work reviewed here shows that cultural associations can be identified in relatively small bodies of text, and can be used to identify patterns at the fine level of granularity of word meaning associations. Roy and Goldwasser (2020) recently combined automated and human coding with embedding analysis to open the way for systematic studies of frames and themes in large volumes of text data.

## DIMENSIONS OF BIAS AS DIRECTIONS IN WORD EMBEDDINGS

Researchers soon discovered that in addition to *distance* (measured by cosine similarity), the geometric property of *direction* in embeddings can be used to study biases. This became apparent after observing how embeddings were able to solve analogy puzzles. Analogies are comparative category–instance associations such as ‘*Paris* is to *France* as *Tokyo* is to *Japan’*. Mikolov et al. (2013) showed that the solution to the analogy ‘*man* is to *woman* as *king* is to ______’ could be determined by adding and subtracting word vectors in a trained embedding: *king* – *man* + *woman* approximates the vector for *queen*. The problem is solved with reference to the directional geometry of the hyperspace: starting at *king*, we move backward (via subtraction) in the direction of *man* and again forward (via addition) into the direction of *woman*; the result is a position within the embedding space that approximates the word *queen*. This procedure can be understood as removing a ‘man’ bias from the word ‘king’ and then adding a ‘woman’ bias. In other words, the directions – or in geometric language, ‘dimensions’ – defined by vectors explicitly *represent* biases.

This realization prompted researchers to develop bias measures based not only on the associated meaning of a single category and trait (i.e., their closeness, as discussed above) but on the comparative judgement of the equivalent trait of a contrasting category. For example, the association between *man* and *surgeon* is equivalent to the association between *women* and *nurse*, and *he* is to *doctors* as *she* is to *midwives* and so on (Bolukbasi et al., 2016). Likewise, Caliskan et al. (2017) investigated gender categories and traits retrieved from Implicit Attitude Tests to show that female names were associated with family words and male names with career words in an embedding created from 840 billion words in text drawn from the internet.

**FIGURE 4:**
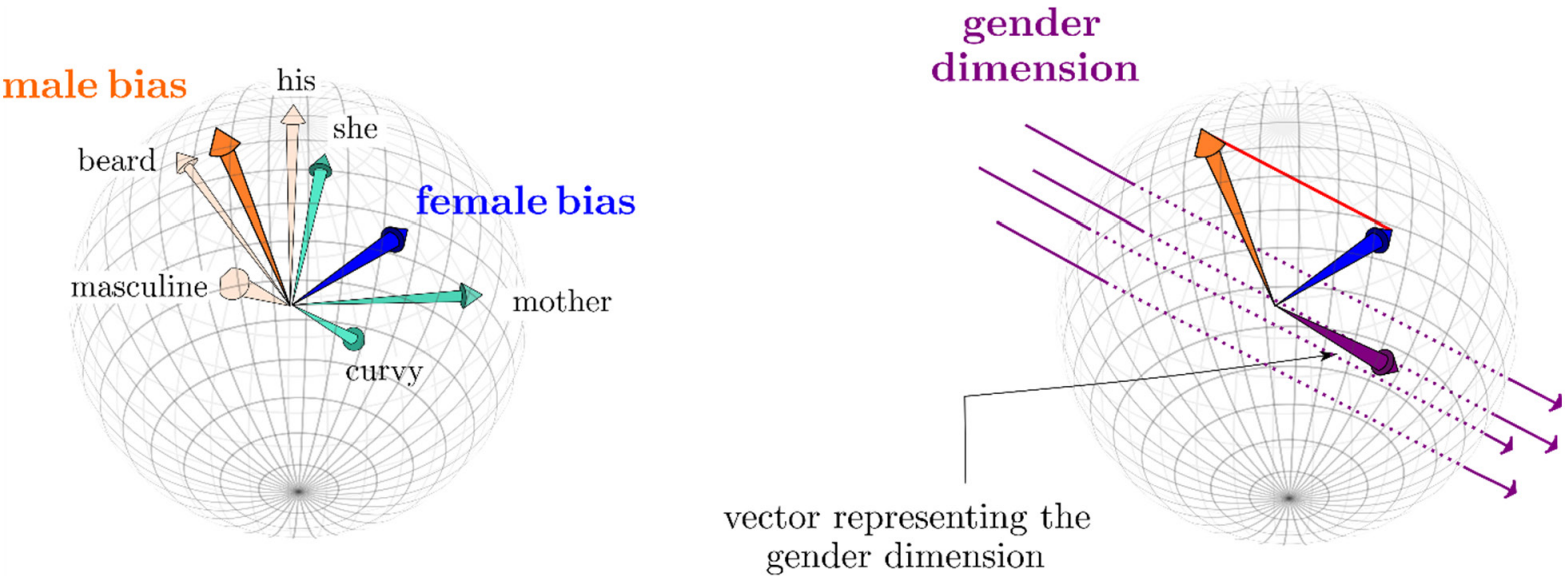
Bias dimensions in word embeddings, simplified to 3‐D. Left: *Male* and *female* bias vectors representing the bias inherent in the words, *masculine*, *beard*, *his* and *she*, *mother*, *curvy* respectively. Right: The difference in the two bipolar bias vectors (centroids) gives rise to another vector representing a bias dimension, which is a direction in the embedding

Bias between comparative categories materializes as a direction in the geometry of an embedding and is determined by computing the difference between word vectors for example, the vector resulting from the subtraction *woman* – *man* defines the direction of a ‘gender bias’. To increase robustness, researchers use sets of words to define the direction of the bias dimension, such as *man*, *he*, *male*, *boy*, *husband* and *son* for the male pole and *woman*, *she*, *female*, *girl*, *wife* and *daughter* for the female pole. Each pole is defined by the centroid of the constituent word vectors, which is itself a vector, and the bias direction is computed as the difference between the centroids. The two centroids will be positioned close to each other in the embedding because the gender words have a very similar use in language, representing human subjects and objects. Analogy tests show that despite their closeness, the orientation of these two poles relative to each other defines a direction in the embedding against which biases can be calibrated with precision. In the process of training, the embedding learns that male and female nouns and pronouns cannot be used interchangeably. Subtracting one polar centroid from the other eliminates all common features of the word meanings that define their closeness in the embedding – that is, their humanness – leaving all that differentiates the male and female poles, namely gender difference in word meaning. The difference between the two centroids is a vector that defines a *gender direction* that runs through the embedding hyperspace (see Figure 4).

Bias dimensions in word embeddings have been likened (Kozlowski et al., 2019) to semantic differential scales used by attitude researchers (cf. Osgood et al., 1957). Both are bipolar evaluative dimensions anchored at opposing poles, and against which evaluations of objects can be gauged as being biased towards one or another pole. The cosine similarity method described above (see Figure 3) is used to quantify the degree to which any word in the embedding projects onto the bias dimension, being orthogonal to the dimension (cosine = 0), perfectly aligned with one or other pole (cosine = −1 or 1) or somewhere in between.

Researchers have constructed bias dimensions to study stereotypes in word embeddings. Kozlowski et al. (2019) used this method to study class and race stereotypes of different music genres in the Google News word embedding, projecting music types (test words) onto rich–poor and white–black cultural dimensions. Using the cosine similarity method, they found that opera and jazz were rich‐biased compared with bluegrass and rap which were poor biased. On the other hand, jazz and rap projected more strongly onto the ‘black pole’ of the race dimension while opera and bluegrass projected more strongly onto the ‘white pole’. A similar approach showed that obesity‐related words carry strong ‘unhealthy’ and ‘female’ bias in New York Times articles (Arseniev‐Koehler & Foster, 2020).

Prejudice towards or against an object can be measured by using the cosine similarity method (Figure 3) to project the word vector for that object onto a bipolar valence dimension anchored in positive and negative words such as good versus bad and love versus hate. Kurdi et al. (2019, Study 3) used this method to determine the extent to which 20 social categories (unemployed, professionals, Muslims, Christians, etc.) were evaluated positively or negatively in 600 billion tokens of online text. They also measured the extent to which these same groups projected onto dimensions of warmth (friendly vs. unfriendly poles) and competence (smart vs. dumb poles). They then used projections to test the hypothesis of the stereotype content model that warmth and competence stereotypes will be uncorrelated or even negatively correlated. In contrast, Kurdi et al. (2019) found these dimensions to be positively correlated both in word embeddings and implicit IAT measures, suggesting that embeddings capture implicit cultural associations in language.

Categories of occupation are biased in familiar ways with *nurse*, *librarian* and *housekeeper* being female and *carpenter*, *mechanic* and *engineer* being male. Garg et al. (2018) examined whether gender biases of these occupations in a Google News trained embedding were associated with real‐world gender discrimination as indexed by employment demographics. Gender bias in their embedding correlated strongly (r = .50) with the relative percentage of women in each category of occupation. Moreover, they found that the overall male bias of occupations decreased over the decades of the twentieth century, corresponding closely with the inclusion of women in these occupation categories over time. Similarly, Jones et al. (2020) found that gendered associations with family (female), career (male), science (male) and arts (female) decreased in English language books published between 1800 and 1990. Importantly, however, bias towards women as homemakers had not eroded despite the profound change to the status of women in the workplace and education over this time.

These results and many others speak to the usefulness of word embedding analysis for investigating cultural biases and testing theoretical hypotheses. They have an almost uncanny power to unearth implicit and familiar stereotypes like the gender associations of occupations and obesity terms, and the racial connotations of music genres. They can expose perplexing ingrained biases in our social world such as the persistent stereotypes of women as homemakers. They can track changes in social biases over time and across contexts. The way in which gendered representations of occupational categories tracked changing gender demographics in the workplace (Garg et al., 2018) suggests that embedding biases may also reflect real‐world discriminatory practices.

## RELIABILITY AND VALIDITY

Word embedding research has developed in the interdisciplinary field of data science which has focused primarily on technology advancement and application. Researchers typically develop tailor‐made tests of embedding quality for their specific application and there is thus ‘still no consensus’ about which methods are best (Bakarov, 2018, p. 1). Nonetheless, there is growing evidence that embedding methods yield reproducible measures of word meanings that correspond closely to conventional meanings. In this section, we will review the methods used to test the quality of embedding bias measures and provide an estimate of the reliability and validity of the measures.

Reliability refers to the reproducibility of measurement, the extent to which ‘scores measured at one time and one place with one instrument predict scores at another time and/or another place and perhaps measured with a different instrument’ (Revelle & Condon, 2019, p. 3). Embedding methods have a number of potential sources of unreliability. Most immediately, the stochastic methods used to train embedding algorithms produce random variation in models trained on the same data. Embedding solutions are also affected by data pre‐processing decisions, the kinds of algorithms and routines of machine learning used and the optimal dimensionality of the word embedding. In addition, measures of closeness between word vectors may be ‘highly sensitive to small changes to the training corpus’ (Antoniak & Mimno, 2018, p. 107).

The effects of these sources unreliably can be tested using different bootstrapping methods. One such test involves subsampling a proportion of the data (typically 90%), and training embedding models on each dataset (Antoniak & Mimno, 2018; Mafunda et al., forthcoming). With large data corpora, the space and processing demand of the method limit the number of bootstrapped embeddings that can be trained. Bias estimates – cosine distance measures and projections onto bipolar dimensions – are computed for each replicated embedding to determine a grand mean and standard deviation. This subsampling bootstrapping method produces more robust bias estimates and allows researchers to judge how features of the embedding depend on the particulars of the data. In addition, design decisions (e.g., are words lemmatized or joined to n‐grams in data pre‐processing, how large is the window of the prediction task, how long do we train for and how large is the space of the embedding) can be varied to identify economical and reliable methods (Mafunda et al., forthcoming). Generally, bootstrapping has shown bias estimates to be remarkably stable and precise (cf. Arseniev‐Koehler & Foster, 2020; Garg et al., 2018; Kozlowski et al., 2019; Rodman, 2020). For example, Arseniev‐Koehler and Foster (2020) found that *boyish* was masculine on each of 25 replications, and on every model, it was less masculine than *tough_guy*. Even small differences in bias can be reliably detected. Kozlowski et al. (2019) found that 90% confidence intervals (based on 20 subsamples) closely hugged the mean cosine closeness estimates gender and class dimension poles – for example, rugged–delicate (.213 ≤ CI ≤ .224), soft–hard (−.168 ≤ CI ≤ −.158) and weak–strong (−.301 ≤ CI ≤ −.287).

The credibility of a method depends on its validity in addition to its reliability. Especially important is a measure's construct validity, and its relationship with the construct of interest (Vazire et al., 2022). Evidence shows that embeddings reflect common sense human understanding of semantics. For example, Roy and Goldwasser (2020) found strong agreement between the way their embedding model and human coders identified the presence of topics in paragraphs of news articles (87%–95% agreement); and Rodman (2020) found *r* = .61 agreement between a ‘gold standard’ model of topic prediction based on human coding and her embedding model.

Embedding bias measures have also been shown to have such subjective face validity. Kozlowski et al. (2019) asked Amazon Turk workers to rate the gender, class and race bias of 54 target words (e.g., boxing, nanny and salad) and found these ratings correlated very strongly with the strength with which these words projected on race (*r* = .70), class (*r* = .52) and gender (*r* = .88) bipolar dimensions of a Google News embedding. Garg et al. (2018) showed that Amazon Turk workers' ratings of the gender stereotypes of occupations correlated strongly with Google New embedding bias measures (*r* ^2^ = .66, *p* < 10^−10^).

Garg et al. (2018) also reported a number of tests of predictive validity. They found that their measure of gender bias of occupations in a Google News predicted the relative proportion of women in those occupations in 2015 (*r* ^2^ = .50, *p* < 10^−10^); and that the change in the relative proportion of women in occupations over decades (between 1910 and 1990) predicted gender bias in embeddings constructed from books published in those decades *(*r^2^ = .24).

Overall, this emerging literature provides grounds for confidence that word embeddings give robust, reproducible and valid estimates of social biases.

**HOW TO TRAIN A WORD EMBEDDING MODEL**

1. *Identify and extract the text data*. The larger the corpus, the better the embedding will perform, and typically word embeddings are trained on millions of documents or sentences. Smaller datasets can be handled by pretraining the model on another, larger corpus, but will inevitably introduce a bias.
2. *Clean and preprocess data*. There are many design choices in this step, for example, whether or not to remove punctuation and stop words, lower‐case, lemmatize or form n‐grams of common word combinations. It should also be decided what ‘units’ are used to train the model, such as full documents (e.g., Wikipedia page), paragraphs or sentences. As a rule of thumb, the pre‐processed data should still be meaningful to a human, since machine learning models are aware of syntax.
3. *Train and store the word embedding*. Modern word embeddings are created by training a machine learning model that solves a text prediction task. Again, there are many design choices such as the window of the prediction, the number of times training sweeps through the dataset or the size of the space for the embedding and choices for technical parameters of the algorithm. Decisions can be supported by monitoring the training error of the prediction error during training (it should decrease and then plateau), or by systematically analysing the final embedding with respect to standard benchmarks, or qualitative analysis of the intended use case.
4. *Use the embedding*. The trained embedding is essentially a list of words and their vectors. These vectors can be used for geometric analysis as described in the main text, for example, to measure distances between two words, or to compute the distance between a word and a dimension in the embedding space. To increase robustness, it is advisable to train several embeddings on bootstrapped datasets, and use average similarities.

One of the most popular ways to train word embeddings in Python is the open‐source library *gensim*, which also offers functionality for data cleaning and analysis. A similar R package is text2vec. While a minimum of coding experience is required for these tools, there is an abundance of beginner's tutorials available online.

## PROSPECTS FOR SOCIAL PSYCHOLOGICAL STUDIES OF EMBEDDING BIAS

In recent years, computerized methods of natural language processing have been touted as tools for conducting qualitative analysis on large representative bodies of text data. In particular, sentiment analysis using the LIWC dictionaries has become influential in psychology and social media analysis (Kerr & Borelli, 2020). However, because these methods rely on predefined meaning dictionaries and a deductive approach to analysis, they are less sensitive to identifying the subtle, context‐dependent biases that social psychologists have foregrounded (see Bruner, 1990; Tileagă et al., 2022).

Word embeddings are well suited to bias analysis of natural language. The embedding methods we have discussed build on the associationist view of bias that is familiar to social psychologists, treating stereotypes as category–trait associations (e.g., immigrants are dangerous; fat is unhealthy) and prejudice as category–valence associations (I dislike immigrants; I hate fat; Greenwald et al., 2002; Stangor, 2016). Word embeddings have an uncanny power to ‘absorb’ biases from culture even when the associations are alluded to or implied but not explicitly stated (Caliskan et al., 2017, p. 1). They can thus be used to detect even subtle biases such as the association of body weight terms (*obese* and *slender*) with women and muscularity terms (*lean*) with men (Arseniev‐Koehler & Foster, 2020).

This work offers opportunities to expand the field of bias research. Social psychologists generally treat bias as mental prejudice: ‘a process that arises – unaided and sometimes contrary to our conscious intentions – from the inner working of our minds’ (Dixon & Levin, 2012, p. 6). The need to incorporate group, collective and ideological levels of analysis is well recognized (see Dixon & Levine, 2012; Tileagă et al., 2022) but we lack methods to do so. For example, Hehman et al. (2018) use multi‐level analysis to show that the police were more likely to use disproportionate lethal force in regions of the United States where individuals on average had higher implicit prejudice. Yet, the collective level of prejudice was determined by aggregating individual‐level data. Word embeddings, by contrast, provide a means of measuring bias in a language community as a whole; and they allow retrospective analysis of bias in historical texts among people who are no longer alive.

The word embeddings reviewed in this article measure biases directly at the collective level of analysis. The meanings embedded in language are shared understandings, communicated in social interaction within communities about issues that matter to them. Word embeddings return information about the language environment in which individuals act and make decisions, judgements and evaluations in different geographical, historical, conversational and ideological contexts. We can measure these biases directly from media, social media and other text data collected from different topics, regions or segments of the population. The studies reviewed here show that embedding methods uncover familiar biases, are sensitive enough to detect biases that are not explicitly stated and can be used to test theoretical hypotheses, for example, about the direction and strength of association between bias dimensions and change over time and context.

Individuals repeat the cultural biases that are embedded in the language they use. However, they are also able to use language to carve out distinct points of view and identify with others to create ‘opinion‐based groups’ (McGarty et al., 2009) that embrace some biases and reject others. Researchers have used computational methods and sentiment analysis to quantify individual stances in controversies on social media (ALDayel & Magdy, 2021). More recently, Rashed et al. (2021) used word embeddings to compute individual stances in polarized discourse, opening the door for embedding research that is able to position individuals in social controversies by the language they use (Schuld et al., 2022). Future research will be able to determine whether traditional measures of individual prejudice predict stances in debate and how these might change over time and across issues.

This research is still in its infancy and there are many questions, limitations and prospects for future development of word embedding analysis and application in social psychology. The relations between words captured in the embedding can be hard to interpret because they are derived from notoriously opaque neural network models in which the context of the word usage is lost. For example, does *first_lady* have a masculine score because the word is closely associated with the word *president*? Future work with contextualized embeddings like BERT models will help to differentiate encoded biases (Kurita et al., 2019) in different contexts (think *dog* in the context of gender and animal talk).

These developments in bias studies are being made by data scientists outside social psychology. Even topics like polarization that are squarely in the field of social psychology are being developed outside the discipline. We hope that this introduction and review has shown the utility of the methods and will motivate social psychologists to experiment with these methods and traverse the technical hurdles.

## AUTHOR CONTRIBUTIONS

**Kevin Durrheim:** Conceptualization; writing – original draft; writing – review and editing. **Maria Schuld:** Conceptualization; visualization; writing – original draft; writing – review and editing. **Martin Mafunda:** Conceptualization; validation; visualization; writing – original draft; writing – review and editing. **Sindi Mazibuko:** Data curation; writing – review and editing.

## CONFLICT OF INTEREST

All authors have no conflict of interest to declare.

## Data Availability

The data that support the findings of this study are available from *Media Monitoring Africa*. Restrictions apply to the availability of these data, which were used under licence for this study. Data are available from the authors with the permission of *Media Monitoring Africa*.

## Footnotes

^1^
The machine learning problem can be formulated in different ways. For example, we try to predict one word from its neighbours, or the neighbours from the one word. BERT machine learning models are able to learn multiple meanings for each word, reflecting their different usages. Embedding analysis differs in the details of how machine learning is designed, but they are similar in that they all produce a vector representation from a prediction task.

^2^
The embedding was trained on Southern African news data provided by Media Monitoring Africa using a *Word2Vec* model from the *gensim* Python library and has dimension *n* = 150.
